# Dichlorido(5,10,15,20-tetra­phenyl­porphyrinato-κ^4^ *N*)anti­mony(V) hemi{di-μ-chlorido-bis­[trichloridoanti­monate(III)]} dichloro­methane monosolvate

**DOI:** 10.1107/S1600536812018351

**Published:** 2012-05-02

**Authors:** Raoudha Soury, Mohamed Sahah Belkhiria, Habib Nasri, Masood Parvez

**Affiliations:** a Département de Chimie, Faculté des Sciences de Monastir, Université de Monastir, Avenue de l’Environnement, 5019 Monastir, Tunisia; b Department of Chemistry, The University of Calgary, 2500 University Drive NW, Calgary, Alberta, Canada T2N 1N4

## Abstract

The asymmetric unit of the title complex, [Sb(C_44_H_28_N_4_)Cl_2_][Sb_2_Cl_8_]_0.5_·CH_2_Cl_2_, is composed of a Sb^V^ complex cation wherein the Sb atom is hexa­coordinated by four N atoms of the pyrrole rings of the tetra­phenyl­porphyrinate (TPP) ligands and two chloride ions, a half di-μ-chlorido-bis­[trichloridoanti­monate(III)] counter-anion and a dichloro­methane solvent mol­ecule. In the cation, the average Sb—N distance is 2.066 (2) Å, while the Sb—Cl distances are 2.3410 (11) and 2.3639 (12) Å. The central unit of the cation, SbN_4_C_20_, is far from being planar, with deviations of atoms from the least-squares plane ranging from −0.110 (4) to 0.124 (4) Å. The Sb—Cl distances in the anion, which is located about an inversion center, lie in the wide range 2.3715 (13)–2.7489 (13) Å, the longest distances being between the Sb and bridging Cl atoms. The crystal structure is stabilized by inter­molecular C—H⋯Cl inter­actions involving the cations, the anions and the solvent mol­ecules. The solvent mol­ecule is disordered over two orientations in a 0.901 (13):0.099 (13) ratio.

## Related literature

For related structures, see: Liu *et al.* (1996 ▶). Ben Moussa *et al.* (2011 ▶).
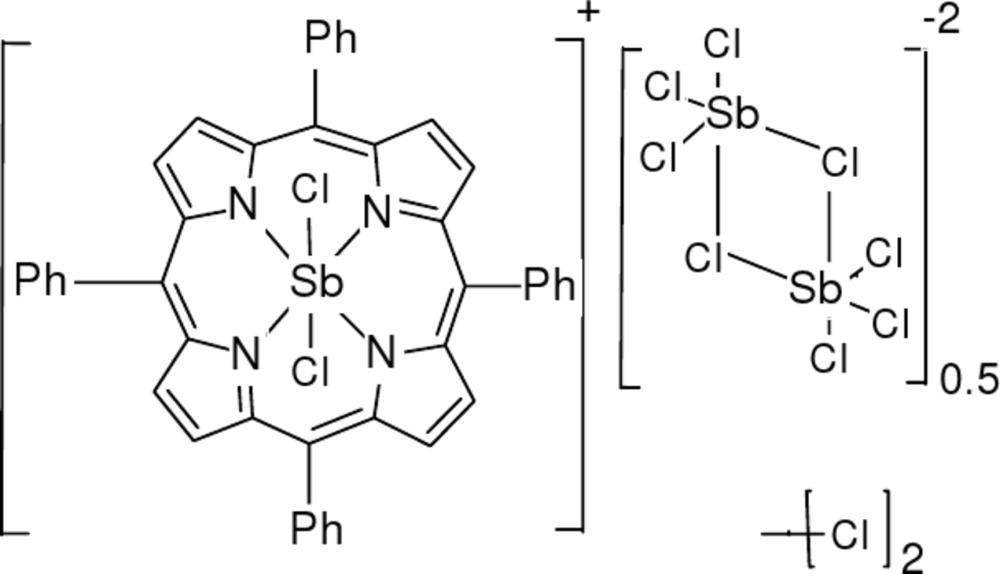


## Experimental

### 

#### Crystal data

[Sb(C_44_H_28_N_4_)Cl_2_][Sb_2_Cl_8_]_0.5_·CH_2_Cl_2_
*M* *_r_* = 1153.83
Triclinic, 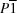

*a* = 11.4488 (2) Å
*b* = 13.3868 (3) Å
*c* = 15.9828 (3) Å
α = 68.3485 (10)°
β = 78.4895 (13)°
γ = 89.8390 (12)°
*V* = 2224.20 (8) Å^3^
*Z* = 2
Mo *K*α radiation
μ = 1.73 mm^−1^
*T* = 173 K
0.16 × 0.14 × 0.11 mm

#### Data collection

Nonius KappaCCD diffractometer
Absorption correction: multi-scan (*SORTAV*; Blessing, 1997 ▶) *T*_min_ = 0.769, *T*_max_ = 0.832
23249 measured reflections
12918 independent reflections
10260 reflections with *I* > 2σ(*I*)
*R*_int_ = 0.039

#### Refinement

*R*[*F* ^2^ > 2σ(*F* ^2^)] = 0.052
*wR*(*F* ^2^) = 0.114
*S* = 1.11
12918 reflections
539 parameters
2 restraints
H-atom parameters constrained
Δρ_max_ = 1.36 e Å^−3^
Δρ_min_ = −0.88 e Å^−3^

### 

Data collection: *COLLECT* (Hooft, 1998 ▶); cell refinement: *DENZO* (Otwinowski & Minor, 1997 ▶); data reduction: *SCALEPACK* (Otwinowski & Minor, 1997 ▶); program(s) used to solve structure: *SHELXS97* (Sheldrick, 2008 ▶); program(s) used to refine structure: *SHELXL97* (Sheldrick, 2008 ▶); molecular graphics: *ORTEP-3 for Windows* (Farrugia, 1997 ▶); software used to prepare material for publication: *SHELXL97*.

## Supplementary Material

Crystal structure: contains datablock(s) global, I. DOI: 10.1107/S1600536812018351/gk2479sup1.cif

Structure factors: contains datablock(s) I. DOI: 10.1107/S1600536812018351/gk2479Isup2.hkl

Additional supplementary materials:  crystallographic information; 3D view; checkCIF report

## Figures and Tables

**Table 1:** Hydrogen-bond geometry (Å, °)

*D*—H⋯*A*	*D*—H	H⋯*A*	*D*⋯*A*	*D*—H⋯*A*
C3—H3⋯Cl6^i^	0.95	2.82	3.515 (4)	131
C8—H8⋯Cl3^ii^	0.95	2.83	3.455 (5)	125
C45—H45*B*⋯Cl4	0.99	2.75	3.562 (7)	140
C31—H31⋯Cl6^iii^	0.95	2.85	3.775 (5)	165

Symmetry codes: (i) 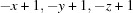; (ii) 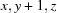; (iii) 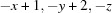.

**Fig. 1.**
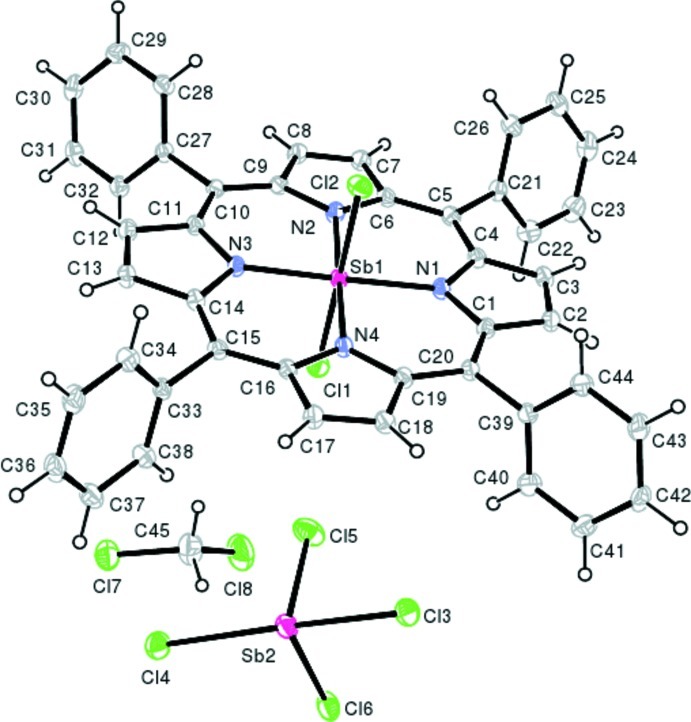
The molecular structure of the title compound with the atom numbering scheme. Displacement ellipsoids are drawn at the 30% probability level. H atoms are presented as small spheres of arbitrary radius and the smaller fraction of the disordered CH2Cl2 has been excluded.

**Fig. 2.**
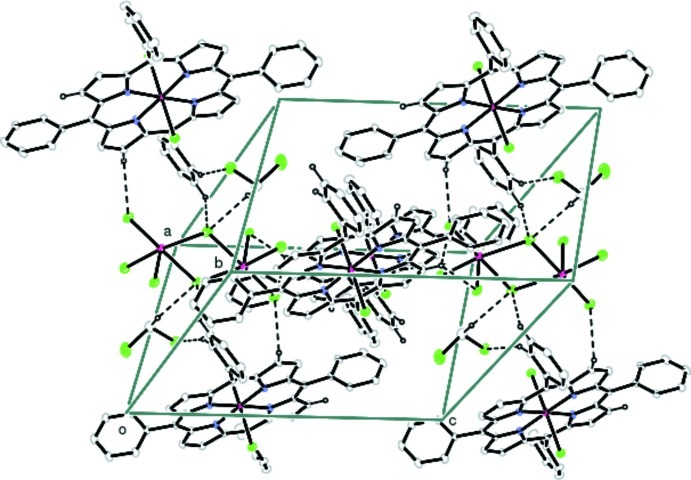
A view of the unit cell packing down the c-axis; H-atoms have been removed for clarity.

## supplementary crystallographic information

### Comment

In continuation of our research on the crystal structures of porphyrin complexes (Ben Moussa *et al.*, 2011) we herein report the synthesis and crystal structure of the title compound.

The asymmetric unit of the title complex is presented in Fig. 1. The molecular dimensions in the cation of the title structure agree very well with the corresponding molecular dimensions reported for a closely related structure (Liu *et al.*, 1996).

The crystal structure is stabilized by C—H···Cl intermolecular hydrogen bonding interactions involving the cations, anions and the solvate molecules (Table 1 & Fig. 2).

### Experimental

The reaction sequence leading to the formation of the title compound is not fully understood at present. SbCl_5_ (1–2 mL) was added to a solution of the porphyrin TPP (500 mg) in pyridine (25 mL) and the resulting solution was refluxed for 1 h. The pyridine and excess SbCl_5_ were removed under low pressure and the resulting purple solid was dissolved in CH_2_Cl_2_ (150 mL). An excess of dimethylglyoxime (80 g) was added *in situ* and the solution was refluxed for 3 h to yield the title compound. The crystal of the title compound were grown by diffusion of hexanes in a CH_2_Cl_2_ solution.

### Refinement

All H atoms were positioned geometrically and refined using a riding model, with C—H = 0.95 and 0.99 Å, for aryl and methylene H-atoms, respectively. The *U*_iso_(H) were allowed at 1.2*U*_eq_(C). The solvate molecule, CH_2_Cl_2_, was disordered over two sites in a 0.901 (13):0.099 (13) ratio. EADP and EXYZ of SHELXL97 (Sheldrick, 2008) commands were used to model the disorder and C45—Cl distances for the smaller fraction were restrained at *DFIX* = 1.77 (1) Å.

### Crystal data

**Table:**

[Sb(C_44_H_28_N_4_)Cl_2_][Sb_2_Cl_8_]_0.5_·CH_2_Cl_2_	*Z* = 2
*M**_r_* = 1153.83	*F*(000) = 1132
Triclinic, *P*1	*D*_x_ = 1.723 Mg m^−^^3^
Hall symbol: -P 1	Mo *K*α radiation, λ = 0.71073 Å
*a* = 11.4488 (2) Å	Cell parameters from 12653 reflections
*b* = 13.3868 (3) Å	θ = 1.0–30.0°
*c* = 15.9828 (3) Å	µ = 1.73 mm^−^^1^
α = 68.3485 (10)°	*T* = 173 K
β = 78.4895 (13)°	Prism, purple
γ = 89.8390 (12)°	0.16 × 0.14 × 0.11 mm
*V* = 2224.20 (8) Å^3^	

### Data collection

**Table:**

Nonius KappaCCD diffractometer	12918 independent reflections
Radiation source: fine-focus sealed tube	10260 reflections with *I* > 2σ(*I*)
Graphite monochromator	*R*_int_ = 0.039
ω and φ scans	θ_max_ = 30.0°, θ_min_ = 2.4°
Absorption correction: multi-scan (*SORTAV*; Blessing, 1997)	*h* = −16→16
*T*_min_ = 0.769, *T*_max_ = 0.832	*k* = −18→18
23249 measured reflections	*l* = −22→22

### Refinement

**Table:**

Refinement on *F*^2^	Primary atom site location: structure-invariant direct methods
Least-squares matrix: full	Secondary atom site location: difference Fourier map
*R*[*F*^2^ > 2σ(*F*^2^)] = 0.052	Hydrogen site location: inferred from neighbouring sites
*wR*(*F*^2^) = 0.114	H-atom parameters constrained
*S* = 1.11	*w* = 1/[σ^2^(*F*_o_^2^) + (0.0155*P*)^2^ + 10.180*P*] where *P* = (*F*_o_^2^ + 2*F*_c_^2^)/3
12918 reflections	(Δ/σ)_max_ < 0.001
539 parameters	Δρ_max_ = 1.36 e Å^−^^3^
2 restraints	Δρ_min_ = −0.88 e Å^−^^3^

### Special details

**Table:**

Geometry. All e.s.d.'s (except the e.s.d. in the dihedral angle between two l.s. planes) are estimated using the full covariance matrix. The cell e.s.d.'s are taken into account individually in the estimation of e.s.d.'s in distances, angles and torsion angles; correlations between e.s.d.'s in cell parameters are only used when they are defined by crystal symmetry. An approximate (isotropic) treatment of cell e.s.d.'s is used for estimating e.s.d.'s involving l.s. planes.
Refinement. Refinement of *F*^2^ against ALL reflections. The weighted *R*-factor *wR* and goodness of fit *S* are based on *F*^2^, conventional *R*-factors *R* are based on *F*, with *F* set to zero for negative *F*^2^. The threshold expression of *F*^2^ > σ(*F*^2^) is used only for calculating *R*-factors(gt) *etc*. and is not relevant to the choice of reflections for refinement. *R*-factors based on *F*^2^ are statistically about twice as large as those based on *F*, and *R*- factors based on ALL data will be even larger.

### Fractional atomic coordinates and isotropic or equivalent isotropic displacement parameters (Å^2^)

**Table:**

	*x*	*y*	*z*	*U*_iso_*/*U*_eq_	Occ. (<1)
Sb1	0.19406 (2)	0.81202 (2)	0.391980 (18)	0.02197 (6)	
Cl1	0.39134 (10)	0.85408 (9)	0.30705 (8)	0.0336 (2)	
Cl2	−0.00618 (10)	0.76926 (10)	0.47554 (8)	0.0357 (2)	
N1	0.2512 (3)	0.7233 (3)	0.5110 (2)	0.0259 (7)	
N2	0.2016 (3)	0.9478 (3)	0.4235 (2)	0.0272 (7)	
N3	0.1322 (3)	0.9002 (3)	0.2750 (2)	0.0240 (7)	
N4	0.1836 (3)	0.6755 (3)	0.3622 (2)	0.0243 (7)	
C1	0.2658 (4)	0.6134 (3)	0.5422 (3)	0.0262 (8)	
C2	0.3058 (4)	0.5848 (4)	0.6263 (3)	0.0286 (9)	
H2	0.3238	0.5148	0.6626	0.034*	
C3	0.3138 (4)	0.6747 (4)	0.6458 (3)	0.0287 (9)	
H3	0.3371	0.6783	0.6985	0.034*	
C4	0.2811 (4)	0.7629 (3)	0.5735 (3)	0.0253 (8)	
C5	0.2790 (4)	0.8702 (3)	0.5671 (3)	0.0266 (8)	
C6	0.2452 (4)	0.9557 (3)	0.4963 (3)	0.0270 (8)	
C7	0.2509 (4)	1.0658 (4)	0.4858 (3)	0.0304 (9)	
H7	0.2770	1.0940	0.5260	0.036*	
C8	0.2125 (4)	1.1233 (3)	0.4082 (3)	0.0296 (9)	
H8	0.2079	1.1992	0.3843	0.036*	
C9	0.1799 (4)	1.0505 (3)	0.3684 (3)	0.0256 (8)	
C10	0.1358 (4)	1.0794 (3)	0.2878 (3)	0.0249 (8)	
C11	0.1119 (4)	1.0082 (3)	0.2460 (3)	0.0258 (8)	
C12	0.0682 (4)	1.0363 (4)	0.1634 (3)	0.0307 (9)	
H12	0.0453	1.1053	0.1293	0.037*	
C13	0.0652 (4)	0.9476 (3)	0.1418 (3)	0.0298 (9)	
H13	0.0400	0.9435	0.0899	0.036*	
C14	0.1066 (4)	0.8609 (3)	0.2112 (3)	0.0256 (8)	
C15	0.1176 (4)	0.7562 (3)	0.2139 (3)	0.0255 (8)	
C16	0.1496 (4)	0.6704 (3)	0.2858 (3)	0.0255 (8)	
C17	0.1498 (4)	0.5608 (3)	0.2936 (3)	0.0293 (9)	
H17	0.1296	0.5340	0.2506	0.035*	
C18	0.1835 (4)	0.5014 (3)	0.3726 (3)	0.0287 (9)	
H18	0.1909	0.4259	0.3948	0.034*	
C19	0.2061 (4)	0.5728 (3)	0.4167 (3)	0.0258 (8)	
C20	0.2459 (4)	0.5441 (3)	0.4992 (3)	0.0252 (8)	
C21	0.3151 (4)	0.8937 (3)	0.6438 (3)	0.0270 (8)	
C22	0.4299 (4)	0.8783 (4)	0.6590 (4)	0.0361 (10)	
H22	0.4882	0.8585	0.6178	0.043*	
C23	0.4598 (5)	0.8918 (4)	0.7342 (4)	0.0440 (12)	
H23	0.5382	0.8795	0.7450	0.053*	
C24	0.3777 (5)	0.9226 (4)	0.7931 (4)	0.0431 (12)	
H24	0.3992	0.9323	0.8441	0.052*	
C25	0.2630 (5)	0.9394 (4)	0.7781 (3)	0.0379 (11)	
H25	0.2058	0.9606	0.8190	0.046*	
C26	0.2313 (4)	0.9255 (4)	0.7031 (3)	0.0321 (9)	
H26	0.1527	0.9377	0.6926	0.039*	
C27	0.1143 (4)	1.1961 (3)	0.2426 (3)	0.0261 (8)	
C28	0.0235 (4)	1.2411 (4)	0.2842 (3)	0.0314 (9)	
H28	−0.0265	1.1978	0.3415	0.038*	
C29	0.0046 (5)	1.3490 (4)	0.2429 (4)	0.0373 (10)	
H29	−0.0579	1.3798	0.2718	0.045*	
C30	0.0776 (5)	1.4120 (4)	0.1590 (3)	0.0357 (10)	
H30	0.0638	1.4856	0.1299	0.043*	
C31	0.1697 (5)	1.3683 (4)	0.1180 (3)	0.0351 (10)	
H31	0.2206	1.4122	0.0614	0.042*	
C32	0.1887 (4)	1.2595 (4)	0.1592 (3)	0.0306 (9)	
H32	0.2519	1.2290	0.1306	0.037*	
C33	0.0998 (4)	0.7337 (3)	0.1318 (3)	0.0268 (8)	
C34	−0.0139 (5)	0.7177 (4)	0.1182 (3)	0.0368 (10)	
H34	−0.0826	0.7209	0.1615	0.044*	
C35	−0.0268 (5)	0.6970 (4)	0.0412 (3)	0.0398 (11)	
H35	−0.1044	0.6856	0.0321	0.048*	
C36	0.0729 (5)	0.6929 (4)	−0.0218 (3)	0.0401 (11)	
H36	0.0637	0.6808	−0.0752	0.048*	
C37	0.1852 (5)	0.7062 (4)	−0.0079 (3)	0.0393 (11)	
H37	0.2534	0.7008	−0.0506	0.047*	
C38	0.1995 (4)	0.7276 (4)	0.0688 (3)	0.0341 (10)	
H38	0.2774	0.7380	0.0778	0.041*	
C39	0.2740 (4)	0.4298 (3)	0.5425 (3)	0.0265 (8)	
C40	0.3759 (5)	0.3939 (4)	0.5034 (3)	0.0389 (11)	
H40	0.4260	0.4414	0.4479	0.047*	
C41	0.4054 (5)	0.2898 (4)	0.5442 (4)	0.0423 (12)	
H41	0.4756	0.2661	0.5166	0.051*	
C42	0.3337 (5)	0.2193 (4)	0.6252 (3)	0.0350 (10)	
H42	0.3551	0.1480	0.6538	0.042*	
C43	0.2311 (5)	0.2538 (4)	0.6636 (3)	0.0382 (11)	
H43	0.1807	0.2056	0.7185	0.046*	
C44	0.2004 (4)	0.3587 (4)	0.6226 (3)	0.0360 (10)	
H44	0.1291	0.3818	0.6495	0.043*	
Sb2	0.45664 (3)	0.49913 (3)	0.13589 (2)	0.03362 (8)	
Cl3	0.40659 (12)	0.35145 (11)	0.29044 (9)	0.0423 (3)	
Cl4	0.51472 (12)	0.65789 (11)	−0.03735 (9)	0.0445 (3)	
Cl5	0.46102 (15)	0.63146 (12)	0.20610 (10)	0.0540 (4)	
Cl6	0.66450 (11)	0.48218 (12)	0.12742 (8)	0.0462 (3)	
C45	0.6101 (7)	0.8636 (5)	0.0311 (4)	0.0613 (17)	
H45A	0.5315	0.8639	0.0703	0.074*	0.901 (13)
H45B	0.6167	0.7909	0.0287	0.074*	0.901 (13)
Cl7	0.6184 (2)	0.9600 (3)	−0.08155 (17)	0.0540 (7)	0.901 (13)
Cl8	0.7249 (3)	0.8909 (4)	0.0805 (2)	0.0829 (11)	0.901 (13)
Cl7'	0.622 (2)	0.9865 (17)	−0.0659 (15)	0.0540 (7)	0.099 (13)
Cl8'	0.7452 (17)	0.850 (4)	0.067 (2)	0.0829 (11)	0.099 (13)
C45'	0.6101 (7)	0.8636 (5)	0.0311 (4)	0.0613 (17)	0.00
H45C	0.5447	0.8644	0.0817	0.074*	0.099 (13)
H45D	0.5919	0.8021	0.0142	0.074*	0.099 (13)

### Atomic displacement parameters (Å^2^)

**Table:**

	*U*^11^	*U*^22^	*U*^33^	*U*^12^	*U*^13^	*U*^23^
Sb1	0.02829 (13)	0.01925 (12)	0.01937 (12)	0.00203 (9)	−0.00664 (10)	−0.00761 (9)
Cl1	0.0301 (5)	0.0343 (6)	0.0330 (5)	−0.0004 (4)	−0.0046 (4)	−0.0099 (4)
Cl2	0.0309 (5)	0.0426 (6)	0.0319 (5)	−0.0004 (5)	−0.0009 (4)	−0.0151 (5)
N1	0.0353 (19)	0.0220 (17)	0.0222 (16)	0.0030 (14)	−0.0101 (14)	−0.0083 (13)
N2	0.0375 (19)	0.0213 (17)	0.0266 (17)	0.0043 (14)	−0.0121 (15)	−0.0108 (14)
N3	0.0327 (18)	0.0194 (16)	0.0205 (15)	0.0027 (13)	−0.0084 (14)	−0.0067 (13)
N4	0.0320 (18)	0.0200 (16)	0.0222 (16)	0.0020 (13)	−0.0090 (14)	−0.0078 (13)
C1	0.031 (2)	0.023 (2)	0.0244 (19)	0.0020 (16)	−0.0070 (16)	−0.0079 (16)
C2	0.037 (2)	0.025 (2)	0.0239 (19)	0.0032 (17)	−0.0099 (17)	−0.0074 (16)
C3	0.035 (2)	0.030 (2)	0.0212 (19)	−0.0002 (17)	−0.0061 (17)	−0.0086 (16)
C4	0.029 (2)	0.025 (2)	0.0225 (18)	−0.0004 (16)	−0.0062 (16)	−0.0094 (16)
C5	0.027 (2)	0.029 (2)	0.0248 (19)	0.0000 (16)	−0.0055 (16)	−0.0113 (17)
C6	0.034 (2)	0.028 (2)	0.0225 (19)	0.0032 (17)	−0.0071 (17)	−0.0136 (16)
C7	0.038 (2)	0.029 (2)	0.030 (2)	0.0048 (18)	−0.0111 (19)	−0.0167 (18)
C8	0.038 (2)	0.020 (2)	0.033 (2)	0.0037 (17)	−0.0095 (19)	−0.0118 (17)
C9	0.033 (2)	0.0184 (18)	0.0237 (19)	0.0029 (15)	−0.0036 (16)	−0.0076 (15)
C10	0.029 (2)	0.0212 (19)	0.0224 (18)	0.0035 (15)	−0.0033 (16)	−0.0068 (15)
C11	0.034 (2)	0.0215 (19)	0.0228 (18)	0.0043 (16)	−0.0084 (17)	−0.0084 (15)
C12	0.037 (2)	0.028 (2)	0.025 (2)	0.0036 (18)	−0.0089 (18)	−0.0063 (17)
C13	0.039 (2)	0.027 (2)	0.025 (2)	0.0018 (18)	−0.0130 (18)	−0.0084 (17)
C14	0.029 (2)	0.025 (2)	0.0214 (18)	−0.0004 (16)	−0.0064 (16)	−0.0076 (15)
C15	0.029 (2)	0.027 (2)	0.0243 (19)	0.0015 (16)	−0.0072 (16)	−0.0132 (16)
C16	0.033 (2)	0.024 (2)	0.0200 (18)	0.0016 (16)	−0.0053 (16)	−0.0087 (15)
C17	0.041 (2)	0.022 (2)	0.027 (2)	0.0014 (17)	−0.0115 (18)	−0.0099 (16)
C18	0.039 (2)	0.0203 (19)	0.026 (2)	−0.0012 (17)	−0.0053 (18)	−0.0082 (16)
C19	0.031 (2)	0.0196 (19)	0.0235 (19)	−0.0011 (15)	−0.0035 (16)	−0.0054 (15)
C20	0.029 (2)	0.024 (2)	0.0221 (18)	0.0016 (16)	−0.0073 (16)	−0.0077 (15)
C21	0.035 (2)	0.023 (2)	0.027 (2)	0.0002 (16)	−0.0129 (17)	−0.0109 (16)
C22	0.033 (2)	0.039 (3)	0.044 (3)	0.0003 (19)	−0.013 (2)	−0.021 (2)
C23	0.047 (3)	0.044 (3)	0.053 (3)	0.005 (2)	−0.030 (3)	−0.022 (3)
C24	0.063 (3)	0.032 (3)	0.041 (3)	−0.005 (2)	−0.026 (3)	−0.013 (2)
C25	0.049 (3)	0.035 (3)	0.030 (2)	−0.005 (2)	−0.005 (2)	−0.015 (2)
C26	0.035 (2)	0.036 (2)	0.028 (2)	−0.0016 (19)	−0.0078 (18)	−0.0158 (19)
C27	0.032 (2)	0.0190 (19)	0.0258 (19)	0.0003 (15)	−0.0065 (17)	−0.0065 (15)
C28	0.036 (2)	0.028 (2)	0.028 (2)	0.0003 (18)	−0.0014 (18)	−0.0105 (17)
C29	0.044 (3)	0.028 (2)	0.045 (3)	0.010 (2)	−0.011 (2)	−0.020 (2)
C30	0.053 (3)	0.019 (2)	0.039 (2)	0.0020 (19)	−0.023 (2)	−0.0088 (18)
C31	0.046 (3)	0.030 (2)	0.025 (2)	−0.007 (2)	−0.009 (2)	−0.0048 (18)
C32	0.036 (2)	0.028 (2)	0.027 (2)	0.0014 (18)	−0.0071 (18)	−0.0089 (17)
C33	0.040 (2)	0.0217 (19)	0.0215 (18)	0.0044 (17)	−0.0109 (17)	−0.0094 (15)
C34	0.042 (3)	0.041 (3)	0.033 (2)	0.008 (2)	−0.013 (2)	−0.018 (2)
C35	0.051 (3)	0.040 (3)	0.040 (3)	0.008 (2)	−0.026 (2)	−0.019 (2)
C36	0.063 (3)	0.033 (2)	0.031 (2)	0.005 (2)	−0.020 (2)	−0.014 (2)
C37	0.054 (3)	0.037 (3)	0.028 (2)	0.003 (2)	−0.007 (2)	−0.015 (2)
C38	0.038 (2)	0.038 (3)	0.029 (2)	0.002 (2)	−0.0072 (19)	−0.0160 (19)
C39	0.034 (2)	0.023 (2)	0.0237 (19)	0.0024 (16)	−0.0058 (17)	−0.0091 (16)
C40	0.042 (3)	0.026 (2)	0.037 (2)	0.0006 (19)	0.004 (2)	−0.0041 (19)
C41	0.044 (3)	0.030 (3)	0.045 (3)	0.011 (2)	−0.002 (2)	−0.010 (2)
C42	0.048 (3)	0.019 (2)	0.035 (2)	0.0048 (19)	−0.014 (2)	−0.0049 (18)
C43	0.044 (3)	0.025 (2)	0.034 (2)	−0.0027 (19)	−0.003 (2)	0.0007 (18)
C44	0.038 (2)	0.027 (2)	0.032 (2)	0.0016 (19)	0.0034 (19)	−0.0037 (18)
Sb2	0.03456 (16)	0.03641 (17)	0.02708 (15)	0.00119 (13)	−0.00417 (12)	−0.00997 (12)
Cl3	0.0442 (7)	0.0393 (7)	0.0340 (6)	0.0009 (5)	−0.0017 (5)	−0.0064 (5)
Cl4	0.0468 (7)	0.0399 (7)	0.0398 (6)	0.0030 (5)	−0.0068 (5)	−0.0082 (5)
Cl5	0.0687 (9)	0.0444 (8)	0.0462 (7)	−0.0090 (7)	0.0052 (7)	−0.0228 (6)
Cl6	0.0370 (6)	0.0648 (9)	0.0278 (5)	0.0030 (6)	−0.0052 (5)	−0.0079 (6)
C45	0.077 (5)	0.052 (4)	0.050 (4)	0.002 (3)	−0.020 (3)	−0.010 (3)
Cl7	0.0503 (8)	0.0634 (13)	0.0424 (9)	0.0025 (9)	−0.0132 (7)	−0.0114 (9)
Cl8	0.0929 (16)	0.108 (3)	0.0576 (12)	0.0136 (16)	−0.0360 (12)	−0.0319 (14)
Cl7'	0.0503 (8)	0.0634 (13)	0.0424 (9)	0.0025 (9)	−0.0132 (7)	−0.0114 (9)
Cl8'	0.0929 (16)	0.108 (3)	0.0576 (12)	0.0136 (16)	−0.0360 (12)	−0.0319 (14)
C45'	0.077 (5)	0.052 (4)	0.050 (4)	0.002 (3)	−0.020 (3)	−0.010 (3)

### Geometric parameters (Å, º)

**Table:**

Sb1—N4	2.061 (3)	C22—H22	0.9500
Sb1—N2	2.063 (3)	C23—C24	1.368 (8)
Sb1—N1	2.070 (3)	C23—H23	0.9500
Sb1—N3	2.070 (3)	C24—C25	1.384 (7)
Sb1—Cl1	2.3410 (11)	C24—H24	0.9500
Sb1—Cl2	2.3639 (12)	C25—C26	1.394 (6)
N1—C4	1.388 (5)	C25—H25	0.9500
N1—C1	1.389 (5)	C26—H26	0.9500
N2—C9	1.386 (5)	C27—C28	1.380 (6)
N2—C6	1.392 (5)	C27—C32	1.393 (6)
N3—C11	1.381 (5)	C28—C29	1.386 (6)
N3—C14	1.384 (5)	C28—H28	0.9500
N4—C16	1.378 (5)	C29—C30	1.390 (7)
N4—C19	1.385 (5)	C29—H29	0.9500
C1—C20	1.386 (6)	C30—C31	1.374 (7)
C1—C2	1.424 (6)	C30—H30	0.9500
C2—C3	1.358 (6)	C31—C32	1.396 (6)
C2—H2	0.9500	C31—H31	0.9500
C3—C4	1.425 (6)	C32—H32	0.9500
C3—H3	0.9500	C33—C38	1.387 (6)
C4—C5	1.402 (6)	C33—C34	1.390 (6)
C5—C6	1.399 (6)	C34—C35	1.392 (6)
C5—C21	1.503 (5)	C34—H34	0.9500
C6—C7	1.420 (6)	C35—C36	1.380 (8)
C7—C8	1.355 (6)	C35—H35	0.9500
C7—H7	0.9500	C36—C37	1.370 (7)
C8—C9	1.431 (6)	C36—H36	0.9500
C8—H8	0.9500	C37—C38	1.397 (6)
C9—C10	1.398 (6)	C37—H37	0.9500
C10—C11	1.405 (6)	C38—H38	0.9500
C10—C27	1.502 (5)	C39—C40	1.382 (6)
C11—C12	1.426 (6)	C39—C44	1.389 (6)
C12—C13	1.356 (6)	C40—C41	1.377 (6)
C12—H12	0.9500	C40—H40	0.9500
C13—C14	1.435 (6)	C41—C42	1.385 (7)
C13—H13	0.9500	C41—H41	0.9500
C14—C15	1.392 (6)	C42—C43	1.374 (7)
C15—C16	1.402 (6)	C42—H42	0.9500
C15—C33	1.502 (5)	C43—C44	1.391 (6)
C16—C17	1.425 (6)	C43—H43	0.9500
C17—C18	1.351 (6)	C44—H44	0.9500
C17—H17	0.9500	Sb2—Cl6	2.3715 (13)
C18—C19	1.431 (6)	Sb2—Cl5	2.4267 (14)
C18—H18	0.9500	Sb2—Cl3	2.4851 (13)
C19—C20	1.400 (5)	Sb2—Cl4	2.7489 (13)
C20—C39	1.493 (6)	C45—Cl8	1.765 (7)
C21—C22	1.384 (6)	C45—Cl7	1.770 (7)
C21—C26	1.391 (6)	C45—H45A	0.9900
C22—C23	1.386 (7)	C45—H45B	0.9900
			
N4—Sb1—N2	178.95 (15)	C19—C20—C39	117.0 (4)
N4—Sb1—N1	89.96 (13)	C22—C21—C26	119.5 (4)
N2—Sb1—N1	89.82 (13)	C22—C21—C5	120.4 (4)
N4—Sb1—N3	90.17 (13)	C26—C21—C5	120.0 (4)
N2—Sb1—N3	90.02 (13)	C21—C22—C23	120.1 (5)
N1—Sb1—N3	178.45 (14)	C21—C22—H22	119.9
N4—Sb1—Cl1	90.38 (11)	C23—C22—H22	119.9
N2—Sb1—Cl1	90.66 (11)	C24—C23—C22	120.7 (5)
N1—Sb1—Cl1	91.30 (11)	C24—C23—H23	119.7
N3—Sb1—Cl1	90.24 (10)	C22—C23—H23	119.7
N4—Sb1—Cl2	89.01 (11)	C23—C24—C25	119.8 (4)
N2—Sb1—Cl2	89.95 (11)	C23—C24—H24	120.1
N1—Sb1—Cl2	89.48 (11)	C25—C24—H24	120.1
N3—Sb1—Cl2	88.98 (10)	C24—C25—C26	120.2 (5)
Cl1—Sb1—Cl2	179.01 (4)	C24—C25—H25	119.9
C4—N1—C1	108.2 (3)	C26—C25—H25	119.9
C4—N1—Sb1	126.0 (3)	C21—C26—C25	119.7 (4)
C1—N1—Sb1	125.8 (3)	C21—C26—H26	120.2
C9—N2—C6	107.8 (3)	C25—C26—H26	120.2
C9—N2—Sb1	125.4 (3)	C28—C27—C32	120.0 (4)
C6—N2—Sb1	126.2 (3)	C28—C27—C10	120.5 (4)
C11—N3—C14	108.3 (3)	C32—C27—C10	119.5 (4)
C11—N3—Sb1	126.1 (3)	C27—C28—C29	120.4 (4)
C14—N3—Sb1	125.5 (3)	C27—C28—H28	119.8
C16—N4—C19	108.4 (3)	C29—C28—H28	119.8
C16—N4—Sb1	125.7 (3)	C28—C29—C30	119.7 (5)
C19—N4—Sb1	125.9 (3)	C28—C29—H29	120.1
C20—C1—N1	126.3 (4)	C30—C29—H29	120.1
C20—C1—C2	126.0 (4)	C31—C30—C29	120.2 (4)
N1—C1—C2	107.6 (4)	C31—C30—H30	119.9
C3—C2—C1	108.2 (4)	C29—C30—H30	119.9
C3—C2—H2	125.9	C30—C31—C32	120.2 (4)
C1—C2—H2	125.9	C30—C31—H31	119.9
C2—C3—C4	108.3 (4)	C32—C31—H31	119.9
C2—C3—H3	125.9	C27—C32—C31	119.4 (4)
C4—C3—H3	125.9	C27—C32—H32	120.3
N1—C4—C5	126.2 (4)	C31—C32—H32	120.3
N1—C4—C3	107.6 (4)	C38—C33—C34	119.6 (4)
C5—C4—C3	126.2 (4)	C38—C33—C15	118.9 (4)
C6—C5—C4	125.5 (4)	C34—C33—C15	121.6 (4)
C6—C5—C21	118.0 (4)	C33—C34—C35	119.9 (5)
C4—C5—C21	116.5 (4)	C33—C34—H34	120.1
N2—C6—C5	125.9 (4)	C35—C34—H34	120.1
N2—C6—C7	108.3 (4)	C36—C35—C34	120.1 (5)
C5—C6—C7	125.8 (4)	C36—C35—H35	119.9
C8—C7—C6	107.9 (4)	C34—C35—H35	119.9
C8—C7—H7	126.1	C37—C36—C35	120.4 (4)
C6—C7—H7	126.1	C37—C36—H36	119.8
C7—C8—C9	108.5 (4)	C35—C36—H36	119.8
C7—C8—H8	125.8	C36—C37—C38	120.1 (5)
C9—C8—H8	125.8	C36—C37—H37	119.9
N2—C9—C10	126.9 (4)	C38—C37—H37	119.9
N2—C9—C8	107.5 (4)	C33—C38—C37	119.9 (5)
C10—C9—C8	125.6 (4)	C33—C38—H38	120.0
C9—C10—C11	125.0 (4)	C37—C38—H38	120.0
C9—C10—C27	116.8 (4)	C40—C39—C44	119.1 (4)
C11—C10—C27	118.2 (4)	C40—C39—C20	119.7 (4)
N3—C11—C10	126.1 (4)	C44—C39—C20	121.2 (4)
N3—C11—C12	108.0 (4)	C41—C40—C39	120.5 (5)
C10—C11—C12	125.8 (4)	C41—C40—H40	119.8
C13—C12—C11	108.1 (4)	C39—C40—H40	119.8
C13—C12—H12	125.9	C40—C41—C42	120.7 (5)
C11—C12—H12	125.9	C40—C41—H41	119.7
C12—C13—C14	108.0 (4)	C42—C41—H41	119.7
C12—C13—H13	126.0	C43—C42—C41	119.1 (4)
C14—C13—H13	126.0	C43—C42—H42	120.4
N3—C14—C15	126.5 (4)	C41—C42—H42	120.4
N3—C14—C13	107.6 (4)	C42—C43—C44	120.6 (4)
C15—C14—C13	125.9 (4)	C42—C43—H43	119.7
C14—C15—C16	125.2 (4)	C44—C43—H43	119.7
C14—C15—C33	117.9 (4)	C39—C44—C43	120.0 (4)
C16—C15—C33	116.8 (4)	C39—C44—H44	120.0
N4—C16—C15	126.7 (4)	C43—C44—H44	120.0
N4—C16—C17	107.6 (3)	Cl6—Sb2—Cl5	90.73 (6)
C15—C16—C17	125.7 (4)	Cl6—Sb2—Cl3	91.77 (4)
C18—C17—C16	108.6 (4)	Cl5—Sb2—Cl3	90.67 (5)
C18—C17—H17	125.7	Cl6—Sb2—Cl4	87.52 (4)
C16—C17—H17	125.7	Cl5—Sb2—Cl4	90.98 (5)
C17—C18—C19	107.7 (4)	Cl3—Sb2—Cl4	178.22 (5)
C17—C18—H18	126.1	Cl8—C45—Cl7	111.5 (4)
C19—C18—H18	126.1	Cl8—C45—H45A	109.3
N4—C19—C20	126.2 (4)	Cl7—C45—H45A	109.3
N4—C19—C18	107.7 (3)	Cl8—C45—H45B	109.3
C20—C19—C18	126.1 (4)	Cl7—C45—H45B	109.3
C1—C20—C19	125.7 (4)	H45A—C45—H45B	108.0
C1—C20—C39	117.2 (4)		
			
N4—Sb1—N1—C4	179.0 (4)	Sb1—N3—C14—C15	0.0 (6)
N2—Sb1—N1—C4	−2.0 (4)	C11—N3—C14—C13	−1.9 (5)
Cl1—Sb1—N1—C4	88.6 (3)	Sb1—N3—C14—C13	179.4 (3)
Cl2—Sb1—N1—C4	−92.0 (3)	C12—C13—C14—N3	1.0 (5)
N4—Sb1—N1—C1	−1.2 (4)	C12—C13—C14—C15	−179.6 (4)
N2—Sb1—N1—C1	177.8 (4)	N3—C14—C15—C16	4.4 (7)
Cl1—Sb1—N1—C1	−91.6 (3)	C13—C14—C15—C16	−174.9 (4)
Cl2—Sb1—N1—C1	87.8 (3)	N3—C14—C15—C33	−172.5 (4)
N1—Sb1—N2—C9	176.1 (4)	C13—C14—C15—C33	8.2 (7)
N3—Sb1—N2—C9	−5.4 (4)	C19—N4—C16—C15	179.2 (4)
Cl1—Sb1—N2—C9	84.8 (4)	Sb1—N4—C16—C15	0.9 (6)
Cl2—Sb1—N2—C9	−94.4 (4)	C19—N4—C16—C17	0.7 (5)
N1—Sb1—N2—C6	5.9 (4)	Sb1—N4—C16—C17	−177.7 (3)
N3—Sb1—N2—C6	−175.7 (4)	C14—C15—C16—N4	−4.9 (7)
Cl1—Sb1—N2—C6	−85.4 (4)	C33—C15—C16—N4	172.1 (4)
Cl2—Sb1—N2—C6	95.3 (4)	C14—C15—C16—C17	173.4 (4)
N4—Sb1—N3—C11	178.9 (4)	C33—C15—C16—C17	−9.6 (7)
N2—Sb1—N3—C11	−0.1 (4)	N4—C16—C17—C18	−0.3 (5)
Cl1—Sb1—N3—C11	−90.7 (3)	C15—C16—C17—C18	−178.8 (4)
Cl2—Sb1—N3—C11	89.9 (3)	C16—C17—C18—C19	−0.2 (5)
N4—Sb1—N3—C14	−2.6 (4)	C16—N4—C19—C20	178.1 (4)
N2—Sb1—N3—C14	178.5 (4)	Sb1—N4—C19—C20	−3.5 (6)
Cl1—Sb1—N3—C14	87.8 (3)	C16—N4—C19—C18	−0.8 (5)
Cl2—Sb1—N3—C14	−91.6 (3)	Sb1—N4—C19—C18	177.5 (3)
N1—Sb1—N4—C16	−179.3 (4)	C17—C18—C19—N4	0.6 (5)
N3—Sb1—N4—C16	2.2 (4)	C17—C18—C19—C20	−178.3 (4)
Cl1—Sb1—N4—C16	−88.1 (3)	N1—C1—C20—C19	−0.7 (7)
Cl2—Sb1—N4—C16	91.2 (3)	C2—C1—C20—C19	179.7 (4)
N1—Sb1—N4—C19	2.6 (4)	N1—C1—C20—C39	176.7 (4)
N3—Sb1—N4—C19	−175.9 (4)	C2—C1—C20—C39	−2.9 (7)
Cl1—Sb1—N4—C19	93.9 (3)	N4—C19—C20—C1	2.3 (7)
Cl2—Sb1—N4—C19	−86.9 (3)	C18—C19—C20—C1	−179.0 (4)
C4—N1—C1—C20	−179.6 (4)	N4—C19—C20—C39	−175.1 (4)
Sb1—N1—C1—C20	0.6 (6)	C18—C19—C20—C39	3.6 (7)
C4—N1—C1—C2	0.0 (5)	C6—C5—C21—C22	−117.5 (5)
Sb1—N1—C1—C2	−179.8 (3)	C4—C5—C21—C22	63.1 (6)
C20—C1—C2—C3	−179.7 (4)	C6—C5—C21—C26	66.0 (6)
N1—C1—C2—C3	0.7 (5)	C4—C5—C21—C26	−113.3 (5)
C1—C2—C3—C4	−1.1 (5)	C26—C21—C22—C23	1.7 (7)
C1—N1—C4—C5	179.6 (4)	C5—C21—C22—C23	−174.8 (4)
Sb1—N1—C4—C5	−0.5 (6)	C21—C22—C23—C24	−1.4 (8)
C1—N1—C4—C3	−0.7 (5)	C22—C23—C24—C25	0.6 (8)
Sb1—N1—C4—C3	179.1 (3)	C23—C24—C25—C26	−0.2 (8)
C2—C3—C4—N1	1.1 (5)	C22—C21—C26—C25	−1.2 (7)
C2—C3—C4—C5	−179.2 (4)	C5—C21—C26—C25	175.3 (4)
N1—C4—C5—C6	0.5 (7)	C24—C25—C26—C21	0.5 (7)
C3—C4—C5—C6	−179.1 (4)	C9—C10—C27—C28	−69.0 (6)
N1—C4—C5—C21	179.8 (4)	C11—C10—C27—C28	111.8 (5)
C3—C4—C5—C21	0.2 (6)	C9—C10—C27—C32	109.4 (5)
C9—N2—C6—C5	−179.4 (4)	C11—C10—C27—C32	−69.7 (5)
Sb1—N2—C6—C5	−7.8 (6)	C32—C27—C28—C29	0.8 (7)
C9—N2—C6—C7	−0.2 (5)	C10—C27—C28—C29	179.3 (4)
Sb1—N2—C6—C7	171.5 (3)	C27—C28—C29—C30	0.1 (7)
C4—C5—C6—N2	3.9 (7)	C28—C29—C30—C31	−1.4 (7)
C21—C5—C6—N2	−175.4 (4)	C29—C30—C31—C32	1.6 (7)
C4—C5—C6—C7	−175.2 (4)	C28—C27—C32—C31	−0.6 (6)
C21—C5—C6—C7	5.5 (7)	C10—C27—C32—C31	−179.1 (4)
N2—C6—C7—C8	−0.4 (5)	C30—C31—C32—C27	−0.6 (7)
C5—C6—C7—C8	178.9 (4)	C14—C15—C33—C38	100.7 (5)
C6—C7—C8—C9	0.8 (5)	C16—C15—C33—C38	−76.4 (5)
C6—N2—C9—C10	179.9 (4)	C14—C15—C33—C34	−80.1 (6)
Sb1—N2—C9—C10	8.2 (6)	C16—C15—C33—C34	102.7 (5)
C6—N2—C9—C8	0.6 (5)	C38—C33—C34—C35	−0.8 (7)
Sb1—N2—C9—C8	−171.2 (3)	C15—C33—C34—C35	−180.0 (4)
C7—C8—C9—N2	−0.9 (5)	C33—C34—C35—C36	−0.3 (8)
C7—C8—C9—C10	179.8 (4)	C34—C35—C36—C37	1.8 (8)
N2—C9—C10—C11	−3.8 (7)	C35—C36—C37—C38	−2.1 (8)
C8—C9—C10—C11	175.4 (4)	C34—C33—C38—C37	0.4 (7)
N2—C9—C10—C27	177.1 (4)	C15—C33—C38—C37	179.6 (4)
C8—C9—C10—C27	−3.7 (7)	C36—C37—C38—C33	1.0 (7)
C14—N3—C11—C10	−174.9 (4)	C1—C20—C39—C40	−104.7 (5)
Sb1—N3—C11—C10	3.8 (6)	C19—C20—C39—C40	72.9 (6)
C14—N3—C11—C12	2.1 (5)	C1—C20—C39—C44	74.1 (6)
Sb1—N3—C11—C12	−179.2 (3)	C19—C20—C39—C44	−108.2 (5)
C9—C10—C11—N3	−2.7 (7)	C44—C39—C40—C41	−1.2 (8)
C27—C10—C11—N3	176.3 (4)	C20—C39—C40—C41	177.7 (5)
C9—C10—C11—C12	−179.2 (4)	C39—C40—C41—C42	−0.1 (8)
C27—C10—C11—C12	−0.1 (7)	C40—C41—C42—C43	1.3 (8)
N3—C11—C12—C13	−1.4 (5)	C41—C42—C43—C44	−1.1 (8)
C10—C11—C12—C13	175.5 (4)	C40—C39—C44—C43	1.4 (7)
C11—C12—C13—C14	0.3 (5)	C20—C39—C44—C43	−177.5 (4)
C11—N3—C14—C15	178.7 (4)	C42—C43—C44—C39	−0.3 (8)

### Hydrogen-bond geometry (Å, º)

**Table:**

*D*—H···*A*	*D*—H	H···*A*	*D*···*A*	*D*—H···*A*
C3—H3···Cl6^i^	0.95	2.82	3.515 (4)	131
C8—H8···Cl3^ii^	0.95	2.83	3.455 (5)	125
C3—H3···Cl3^i^	0.95	2.99	3.528 (5)	117
C45—H45*B*···Cl4	0.99	2.75	3.562 (7)	140
C31—H31···Cl6^iii^	0.95	2.85	3.775 (5)	165
C32—H32···Cl4^iii^	0.95	2.90	3.512 (5)	123
C32—H32···Cl7^iii^	0.95	3.19	4.076 (6)	155

Symmetry codes: (i) −*x*+1, −*y*+1, −*z*+1; (ii) *x*, *y*+1, *z*; (iii) −*x*+1, −*y*+2, −*z*.
